# Miscellaneous Observations on the Medical and Surgical Uses of Cold Water

**Published:** 1786

**Authors:** Nicholas Chavasse

**Affiliations:** Surgeon, at Walsall, in Staffordshire, and Member of the Corporation of Surgeons of London


IV. *Miſcellaneous Obſervations on the Medical and Surgical Uſes of cold Water. By Mr.* Nicholas Chavaſſe, *Surgeon, at Walſall, in Stafford-ſhire, and Member of the Corporation of Surgeons of London.*

CASES of obſtinate and chronic vomiting, which ſeem to depend ſimply on a morbid bid irritability of the ftomach, often occur in practice, and when violent, are fufficient caufe of uneafinefs to the practitioner, as well as of diftrefs to the patient. It is a complaint feldom fatal in itfelf, yet eventually lays the foundation of various chronic affections.

It is cuftomary to adminifter an emetic when we hear patients complaining of naufea and vomiting; and where thefe fymptoms originate from an accumulation of irritating matter in the ftomach, the practice generally proves fuccefsful; yet cafes do occur where this propenfity to vomit bids defiance to evacuants of every denomination, as well as to the other modes of relief commonly adopted in fuch cafes.

In complaints of this kind, after evacuations have been liberally ufed, and there has been every reafon to believe all irritating matter difcharged, and the difeafe has been evidently not fymptomatic, or the effect of inflammation, I have often been foiled in removing it fo fpeedily as I at firft expected. This led me to the ufe of the following plan, among many others, and its efficacy I firft proved in the following cafe:

The patient was a man of a ftrong, athletic habit, abftemious, and of little irritability. He had had enjoyed a good ſtate of health till this attack of nauſea and vomiting, for which he could aſſign no other cauſe than that of having eaten a few muſhrooms; for ſoon after he was ſeized with nauſea, vertigo, and pain in the ſtomach at intervals. Vomiting was induced by the exhibition of a few grains of pulv. ipecac. and encouraged by a liberal ſupply of carduus tea. On the following day he took a purge, part of which was rejected; yet enough paſſed into the bowels to evacuate their contents. From this period the vertigo and pain in the ſtomach ceaſed; but the retching ſtill harraſſed the patient with increaſed violence. At his own requeſt we now had recourſe to a more powerful emetic, and an antimonial one was adminiſtered in frequent and divided doſes. This was rough in its operation, and only procured a temporary ſuſpenſion of the diſeaſe. The ſtomach was now diſguſted with every thing, except cold water, and this did not always remain down. Gruel, broth, &c. when of the leaſt warmth, excited a paroxyſm of vomiting equal in violence to that produced by an active emetic. The ſtomachic plaſter, and afterwards a bliſter, were applied to the region of the ſtomach, but without any good effect: opium was of no ufe, though given by itfelf in various forms, and combined with other medicines; nor was any benefit obtained from the faline draught taken in a ftate of effervefcence, Colombo root, &c. — The difeafe, at the expiration of three weeks, was worfe than at firft, and the patient much emaciated. The complaint had been at firft apparently brought on by the eating of mufhrooms; yet, from the repeated ufe of emetics and purges, thefe muft have been evacuated. There was no reafon to believe it fymptomatic, or to fufpect any morbid affection of the abdominal vifcera. The difeafe was probably fupported by a topical affection of the ftomach, furcharged with irritability: the cure at leaft authorifes the fuggeftion. Cold has a powerful tendency to remove this, when exceffive; and, upon the principle of its being a fedative, I made ufe of it in the prefent cafe.

It may be worth mentioning, that the patient occafionally complained of an uncommon fenfation of heat at the ftomach during his illnefs, and this he had learned to mitigate by expofing the region of the difeafed organ to the influence of cold air. He was now directed to take half a drachm of the bitter purging falt in half a pint a pint of cold water, while in the act of solution, and repeat it every second hour; and towels, wet with water, artificially rendered colder than in its natural state, were applied to the region of the stomach, and frequently renewed. The patient was soon sensible of relief, the vomiting abating in its violence, and the sensation of accumulated heat quickly becoming more tolerable. On the third day he was so much better as to take a short journey, but returned home with a slight attack of his disease, owing to the officious hospitality of his friends; but this was permanently removed by having recourse to the old plan.

There was no reason, in this case, to assign the heat of the stomach to inflammation; for gastritis never continues so long, and is ever accompanied with acute pain. It is remarkable that the solution invariably suppressed the vomiting, without diminishing the sensation of heat; and that the external application of cold mitigated this sensation, without alleviating the propensity to vomit.

I may have been too prolix in relating this case; yet, in defence of this, I would urge, that it is often from a combination of minute circumstances (separately of little weight) that we are enabled to deduce important facts. The premifes may appear uninterefting, but the conclufion may be of confiderable weight.

As the ftomach has a motion analogous to that of the inteftines, may not a fufpenfion or inverfion of that motion be often a caufe of this difeafe? If this is allowed, are we not adopting a rational plan, by encouraging the ftomach to protrude its contents into the duodenum, and preparing our patient for the exhibition of fuch medicines as are calculated to give energy and health to the digeftive organs?

The judicious application of this fimple element in various difeafes, dependent on irritability and fpafm, will often prove of more real fervice than our boafted antifpafmodics, tonics, and fedatives. In cafes of hyfteria, cold water fuddenly applied to the region of the ftomach has an immediate and powerful tendency to remove that fuffocating diftention attendant upon the hyfteric paroxyfm.—The fedative, ftudious, and valetudinary are often afflicted with violent and alarming head-ach. This difeafe is difficult to palliate when prefent, and its recurrence not eafily obviated. For more than two years I have been feverely handled, at times, by this complaint. After trying various means without benefit, I have now learned to mitigate, and finally to remove it, by the application of cold water to the head.

There is an affection to which hysteric persons are subject, and which, like their other complaints, is more disagreeable than dangerous: I mean a vermicular, crawling sensation in different parts of the body, but more especially in the legs and arms. The sudden application of cold water to the part affected generally removes it; and should be had recourse to when the use of the cold bath is not complied with. Its efficacy in alleviating many of the symptoms of this disease depends on its being suddenly applied.—In retention of urine, cold water dashed on the *regio pubis* will often supersede the use of the catheter: indeed I fear much injury often ensues from the precipitate and indiscriminate use of that instrument. It is not presumed that it can be found serviceable in urinary suppressions depending on inflammation, schirrosity, or mechanical obstructions: it is only recommended in simple retention, dependent on a deficient contraction of the bladder upon its contents. I lately had the pleasing satisfaction of seeing a decided proof of its utility in the case of a person who had made no water for five and thirty hours. The patient could aſſign no other cauſe for this inability than his having diſregarded nature's ſolicitations. Upon examination, I found a large tumor above the pubis. As he was in violent pain, and the pulſe was accelerated, he was bled. Terebinthinate and opiate clyſters were injected, and he was put into a warm bath. It is true he took no ſtimulating medicines by the mouth; for I was apprehenſive they would ſerve only to augment the flow of water to the bladder. The diſeaſe continued, however, in ſpite of our efforts; for theſe did not produce the evacuation of a drop of water. It now occurred to me, that the contractile power which cold has upon the ſkin might, in this caſe, be advantageouſly extended to the bladder, and ſo the event proved; for, after a few trials, the urine came away *guttatim*, and, as the action of the bladder increaſed, was diſcharged freely.

I remember to have ſeen, when in Jamaica, a caſe of inteſtinal hæmorrhage that proved fatal. Several aſtringent and ſtyptic medicines were given by the direction of a ſurgeon of eminence in that iſland; yet nothing ſeemed ever to check the diſcharge of pure blood, and at length the patient ſunk under a gradual hæ-

morrhage.

morrhage. Upon opening the body, I diſcovered nothing that could mechanically produce a rupture of an artery in the large arch of the colon, from which the hæmorrhage ſeemed to have proceeded. Had water in its coldeſt ſtate been injected *per anum*, it might have been the means of reſcuing a deſerving man from an untimely death.

The vegeto-mineral water of Monſieur Goulard is a faſhionable application of the day; but I am inclined to think with ſome late writers*, that praiſes have been laviſhed upon the extract of lead, when they ſhould have been beſtowed on the water that enters the compoſition of the vegeto-mineral water. In my own practice I have been able to accompliſh as much with water alone, as when combined with the extract of lead. It will be urged, perhaps, that ſome additional efficacy may be derived from the increaſed degree of cold produced by their mixture; but, till this increaſe of cold is demonſtrated, I muſt withhold my aſſent to increaſe of efficacy: for, after repeated trials with Fahrenheit's thermometer, I have not

* See, in particular, an ingenious Eſſay on the Theory of the Production of Animal Heat, by Mr. Rigby, p. 121.

been been able to prove that fuch an effect is pro-duced. We are not to conclude from this that it is a medicine of negative powers; for I remember to have feen a tranfient paralyfis conftantly follow its application. This evi-dently proves that it has a powerful effect on the fentient powers; but this effect is more likely to occafion difeafe than to accelerate a cure; for, in a furgical point of view, what practitioner would think of expediting the heal-ing procefs by reducing the vital energy below the ftandard of health? All that he requires is to moderate it when exceffive, and he may rea-dily arrive at this *ne plus ultra* by the ufe of cold water. This, at leaft, I have repeatedly experienced in different attacks of local inflam-mation; but in no cafe is its falutary power more ftriking than in thofe affections produced either by actual or potential heat. Seceffion of pain immediately follows its application: it moderates the inflammation, and, if the epi-dermis is fortunately left entire, fpeedily re-duces the parts to a ftate of original foundnefs by the defirable procefs of the firft intention. — I do not from this conclude, that from its ufe alone we fhall be able to cicatrize parts in a ftate of fuppuration; but even here it will be benefi-cial cial in diluting the acrimony of the difcharge, in wafhing away irritating matter, and, if affifted by a proper degree of compreffion, will certainly expedite the cure.

Perhaps it will be found, upon inquiry, that feveral fprings, celebrated for their healing powers, owe their celebrity folely to the cleanfing effects of their waters. It is too much the practice to apply hot cataplafms indifcriminately in every cafe of recent fracture, whether fimple or compound, whether the fubject be of a relaxed or rigid fibre. This irrational treatment is frequently productive of additional pain, and, in moft cafes, eventually retards the cure. The hiftory of the following cafe ftrongly tends to confirm this idea: — The patient was a young girl, of an irritable habit and lax fibre; the fracture was fimple, and confined to the fibula. The practitioner who ufually attended the family applied a hot cataplafm to the limb, and although the inflammation and tenfion evidently increafed under its ufe, he had recourfe to a fomentation. The inflammation and tumefaction now befet the whole limb, and the patient experienced fuch an intolerable fenfation of heat as induced delirium. Upon being confulted, I recommended the application

 of of cold water, and to aſſiſt its evaporation from the inflamed ſurface by every poſſible means. In leſs than two hours this morbid accumulation of heat began to leſſen; and, at the expiration of thirty, the patient was free from much pain.

Is not the application of heat to recent fractures the moſt likely means to induce that very inflammation which it is the ſurgeon's duty to obviate? Is not the application of hot ſteam likely to invite a flux of humours to parts already prediſpoſed to accumulation by the mechanical powers of irritation?

It is indeed ardently to be wiſhed that this practice, ſo pernicious in its conſequences, were aboliſhed. From the opportunities that have offered in the courſe of my own practice, I can aſſert, that the ſedative powers of cold water have been ſufficient to reduce the parts to that ſtate of inflammation abſolutely neceſſary to conſolidate the union of the fractured bones; and I believe that cold, judiciouſly applied, in many caſes dependent on local irritability, or upon local accumulation of animal heat, will often produce the moſt ſalutary effects, when other more boaſted and elaborate remedies will prove nugatory and inefficient.

