# Errata

**Published:** 1787

**Authors:** 


### ERRATA.

Vol. VII. page 128, for *fedative*. read *fedentary*.

